# Local professionals’ perceptions of health assets in a low-SES Dutch neighbourhood: a qualitative study

**DOI:** 10.1186/s12889-017-4555-6

**Published:** 2017-07-12

**Authors:** Lea Den Broeder, Ellen Uiters, Aafke Hofland, Annemarie Wagemakers, Albertine Jantine Schuit

**Affiliations:** 1 Centre for Nutrition, Prevention and Health Services, National Institute for Public Health and the Environment, Bilthoven, The Netherlands; 2 School of Sports and Nutrition, Amsterdam University of Applied Sciences, Amsterdam, The Netherlands; 3 Wageningen University & Research Centre Health and Society, Wageningen, The Netherlands; 4 Faculty of Earth and Life Sciences, VU University, Amsterdam, The Netherlands

**Keywords:** Professionals, Perceptions, Neighbourhood, Low-SES, Asset-based approach, Positive health

## Abstract

**Background:**

Asset-based approaches have become popular in public health. As yet it is not known to what extent health and welfare professionals are able to identify and mobilise individual and community health assets. Therefore, the aim of this study was to understand professional’s perceptions of health and health assets.

**Methods:**

In a low-SES neighbourhood, 21 health and welfare professionals were interviewed about their definition of health and their perceptions of the residents’ health status, assets available in the neighbourhood’s environment, and the way residents use these assets. A Nominal Group Technique (NGT) session was conducted for member check. Verbatim transcripts of the semi-structured interviews were coded and analysed using Atlas.ti.

**Results:**

The professionals used a broad health concept, emphasizing the social dimension of health as most important. They discussed the poor health of residents, mentioning multiple health problems and unmet health needs. They provided many examples of behaviour that they considered unhealthy, in particular unhealthy diet and lack of exercise. Professionals considered the green physical environment, as well as health and social services, including their own services, as important health enhancing factors, whereas social and economic factors were considered as major barriers for good health. Poor housing and litter in public space were considered as barriers as well. According to the professionals, residents underutilized neighbourhood health assets. They emphasised the impact of poverty on the residents and their health. Moreover, they felt that residents were lacking individual capabilities to lead a healthy life. Although committed to the wellbeing of the residents, some professionals seemed almost discouraged by the (perceived) situation. They looked for practical solutions by developing group-based approaches and supporting residents’ self-organisation.

**Conclusions:**

Our study shows, firstly, that professionals in the priority district Slotermeer rated the health of the residents as poor and their health behaviour as inadequate. They considered poverty and lack of education as important causes of this situation. Secondly, the professionals tended to talk about barriers in the neighbourhood rather than about neighbourhood health assets. As such, it seems challenging to implement asset-based approaches. However, the professionals, based on their own experiences, did perceive the development of collective approaches as a promising direction for future community health development.

**Electronic supplementary material:**

The online version of this article (doi:10.1186/s12889-017-4555-6) contains supplementary material, which is available to authorized users.

## Background

In public health, ‘asset-based’ approaches have become increasingly popular as a potential way to improve health at the community level [1–3]. Such approaches focus on linking up with both individual and community capacities and capabilities, including (creating) important opportunities for community health in the neighbourhood’s social and physical environment, for example walkability of the local spatial design, educational and social facilities, or employment options. Asset-based approaches are developed to be applied in communities; hence instead of focusing on individuals, they work primarily on the community level [1, 3]. For health and welfare professionals working in communities applying this asset-based approach means that they need to be able to identify, find and mobilise these assets, in coordination with the residents. This may not be unproblematic, as many professionals have been trained and have worked in a medically oriented system that traditionally emphasised delivering services to ‘passive’ citizens [4, 5].

However, two important national policy developments in the Netherlands urge professionals to more closely link up to the discourse of asset-based approaches. Firstly, a policy transformation regarding public health and welfare takes place that may be understood as a shift from ‘caring for residents’, to stimulating residents’ own capacities to look after their own, and each other’s health and wellbeing [6]. Policy makers are assuming that this policy shift will help improve the population’s health and, above all, lead to lower health care costs. Currently experiments are carried out to test this assumption (see, for example, [7, 8]). In relation to this development, Dutch policy makers are embracing the new concept of ‘positive health’ as the individual capacity to self-manage and being able to cope with setbacks and difficult circumstances. ‘Positive health’ was introduced by Huber et al. [9] arguing that the usual focus on ‘disease’ and ‘disability’ is no longer appropriate in modern age where early detection leads to ‘ill’ people without symptoms, and where many people can live a good life with a well-managed chronic condition. Positive health gets a lot of attention in the Netherlands [10].

A second important policy development is the so-called ‘priority neighbourhoods’ policy. The 40 most disadvantaged neighbourhoods in the Netherlands, diagnosed to have an accumulation of health and social problems, receive special governmental support (including finances) to develop and carry out integrated programmes to upgrade the neighbourhood. Recent evaluation has indicated that the programmes implemented under this policy in these ‘priority districts’ have benefited, or have the potential to benefit the health of the communities in these areas by creating a more health-enhancing physical and social environment [11–13], but not all interventions by themselves generated the expected health impact. For example, investment in green areas as a stand-alone intervention did not seem to have a meaningful influence on the health status of the residents [14]. Although this policy is not explicitly labelled as ‘asset-based’, it can be understood to link up with this approach, seeking to develop opportunities for better health in disadvantaged communities.

In sum, the promise of asset-based approaches at the community level and recent policy developments urge professionals to work according to these approaches, in particular in the ‘priority districts’. However, as far as known, in practice asset-based approaches are only applied occasionally. In order to understand why this - apparently - is such a challenge for professionals, the aim of this paper is to report on the perception of professionals, based in a ‘priority district’, on health, neighbourhood assets and residents’ capacities to create and maintain good health. The following two research questions were addressed:

- What is the professionals’ perception of health and of residents’ health status?
- What is the professionals’ perception of available health assets in the neighbourhood and the way residents use these assets?

## Methods

### Setting and recruitment of professionals

The setting for this study is the Amsterdam neighbourhood of Slotermeer (Table 1) [15–18]. Slotermeer is one of the ‘priority neighbourhoods’ included in the national improvement program.

**Table 1:** Background details about Slotermeer neighbourhood

Amsterdam-Slotermeer (26.000 residents) is located in the city district Nieuw-West, on the west side of Amsterdam outside the city centre. It is a so-called ‘garden suburb’ built after the Second World War, implementing the vision of the urbanist Van Eesteren^[15]^ with plenty of light, air and space; part of Slotermeer is a protected city view. Slotermeer is considered as a problem neighbourhood. Statistics for several health determinants, like smoking, unhealthy diet and lack of physical exercise, as well as for health parameters like obesity, diabetes, depression and suicide compare negatively to those in other parts of the city^[16]^. 29% of the residents reports severe loneliness; in Amsterdam as a total the severe loneliness rate is 11%^[17]^. The population includes more families and children, compared to other city districts. The 23% proportion of residents over 55 is similar to the Amsterdam average. The neighbourhood faces severe socioeconomic problems like high unemployment and debts, and residents rate the liveability as low in comparison to residents in other neighbourhoods^[18]^. Three out of ten households (28%) have a low income and a breadwinner with low educational level, which is 15% in Amsterdam over-all^[17]^. However, the neighbourhood has many active residents, amongst others in the highly successful ‘neighbourhood living rooms’ where residents meet for social activities. The cultural composition of the population is very diverse and over 60% are ‘Amsterdammers’ of non-western origin^[17]^.	

In Slotermeer, we interviewed a varied group of professional health and care workers, in order to obtain a broad range of different visions and approaches (purposive sampling). We selected names of professionals by using the categories ‘health’ and ‘welfare’ of the social map of the neighbourhood [19]. In addition, we applied the ‘snowball’ method, asking professionals we knew in the neighbourhood to provide contacts. In total 45 professionals were invited to participate in the study by e-mail and telephone follow-up. Criteria for inclusion of professionals were that the professionals 1) had worked in the neighbourhood of Slotermeer for at least one year and 2) had a good command of Dutch.

Twenty one professionals volunteered to participate in the study (Table 2). All of them fulfilled the inclusion criteria. The other 24 professionals were unable to take part because of either lack of time or unavailability during the interview period. Seven of the professionals who participated in the interviews also participated in a Nominal Group Technique (NGT) session [20]. This method allows a group of people to creatively think up and rank possible decisions or solutions to a problem. A NGT session consists of individual brainstorming, joint listing of all alternative decisions or solutions identified by the participants, subsequent discussion of each item and one or more rounds of individual rating, after which the total score per item is calculated. Fourteen professionals indicated that they did not have time or interest to participate in the NGT session.

**Table 2:** Overview of interviewed professionals

Type of organisation	Professional *N* = 21	Role in health and welfare system
GP Practice	General practitioner (GP) (*n* = 2)	Medical doctor trained for primary and family care. Provides primary medical services and is gatekeeper to hospital and specialist care. To provide out of office care country-wide, GPs cooperate in regional out of office GP posts. Coverage 100% under the (mandatory) health insurance system.
	Doctor’s assistant (*n* = 2)	Trained assistant to the GP, providing front office services and assisting in care provision. Coverage 100% under the (mandatory) health insurance system.
	GP nurse (*n* = 3)	Doctor’s assistant with extended training, providing counselling and mentoring to patients with chronic diseases like diabetes or COPD. Works under the supervision of the GP. Coverage 100% under the (mandatory) health insurance system. Coverage 100% under the (mandatory) health insurance system.
Other health care organisation	Dietician (*n* = 2)	Provides services in primary care for patients with specific dietary needs as well as preventive services. Coverage under the (mandatory) health insurance system for a limited number of consultations per patient.
	Community nurse (*n* = 2)	Provides home based care services to patients in their personal living environment, e.g. home based wound care, care for terminally ill etc. Coverage 100% under the (mandatory) health insurance system.
	Youth health care doctor (*n* = 1)	Provides preventive services for youth 0–18. Refers children/youth with health or other problems to GP or specialised services. Coverage 100% by all Dutch municipalities under the Public Health Act.
	Physiotherapist (*n* = 1)	Provides physiotherapy as a primary care service. Coverage under the (mandatory) health insurance system for a limited number of consultations per patient.
	Midwife (*n* = 1)	Provides pregnancy care and counselling and birth care including both home and hospital birth. Is a recognised medical professional. Refers to gynaecologist in case of complications. Coverage 100% under the (mandatory) health insurance system.
Welfare organisation	Family coach (*n* = 1)	Provides family-based coaching services. Coverage under the (mandatory) health insurance system for a limited number of consultations per client.
	Community worker (*n* = 2)	Provides community services, building and supporting community groups. Financed through municipal budget for social services. Stationed in community centre.
	Youth worker (*n* = 2)	Provides community services focused on youth. Financed through municipal budget for social services. Usually stationed in community centre.
	Volunteer coordinator (*n* = 1)	Provides support for volunteers and volunteer services in the community, including volunteers in social support, welfare work, elderly people’s care, etc. Financed through municipal budget for social services.
	Social worker (*n* = 1)	Provides support for families and individuals with a variety of challenges including financial, social and mental problems. Refers to care system if needed. Financed through municipal budget for social services.

### Interviews and Nominal group technique

We used a semi-structured interview protocol. In the first part of the interview, addressing the first research question, the professionals were asked how they defined health, how they perceived the health status of residents, and what they thought residents and professionals could do to maintain good community health or improve it. Examples of questions were: ‘What is health, according to you?’, ‘How healthy are the people in Slotermeer?’ and ‘What possibilities do residents have, to do something about their health?’.

The second part concerned the assets for health in the living environment perceived by the professionals. These were described broadly as ‘features of the neighbourhood that provide possibilities for health’ as the term ‘assets’ has no Dutch synonym. To illustrate what ‘assets’ are and to challenge the professionals to think about a broad range of neighbourhood assets we used the ‘Egan wheel’ [21] which contains seven neighbourhood dimensions. Table 3 contains a summary of the interview protocol.

**Table 3:**
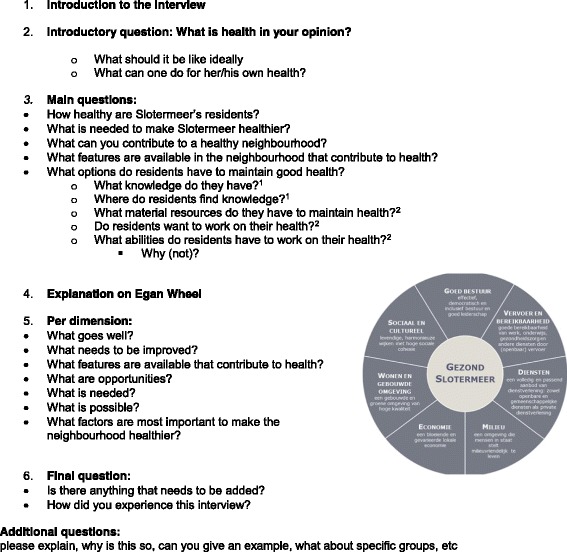
Summary of interview protocol

Subsequently we organised a member check with the interviewed professionals: we conducted a NGT session to verify first analysis results [22]. In this session, we shared the interview results with the professionals. Subsequently they were invited to comment. Then, the NGT technique was applied to answer the central question: “What are best options in the neighbourhood environment that could contribute to improve the community’s health?”.

### Codebook development and analysis

**Fig. 1:**
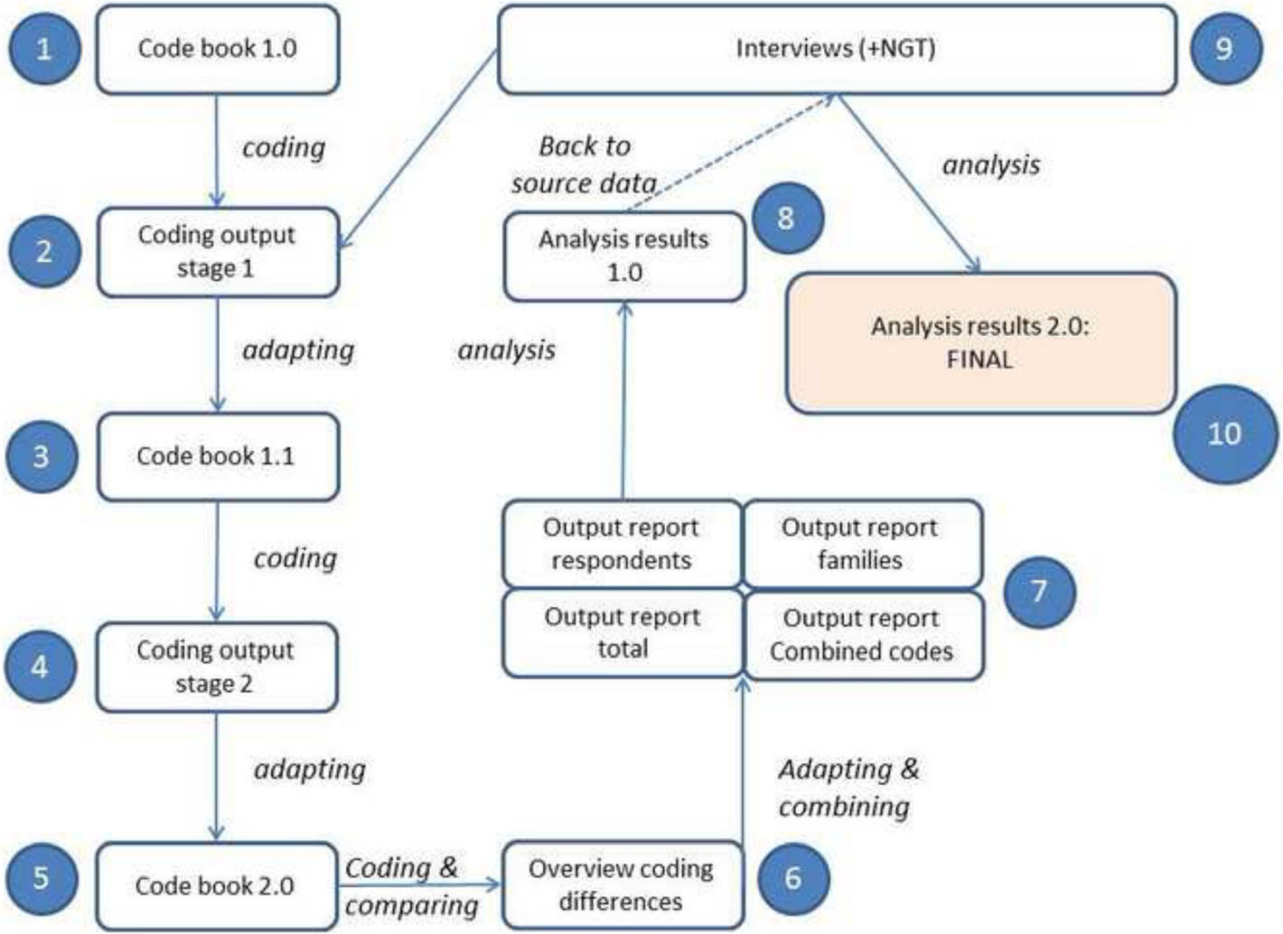
Overview of analysis process

The development of the codebook and analysis of data was an iterative process involving 10 steps (Fig. 1).

An initial codebook (version 1.0) was developed (step 1) based on concepts used in the research questions (e.g. health, health status) and the methods (e.g. wheel of Egan). The codebook has been refined during the coding process: after coding 1 interview transcript (steps 2 and 3), and after coding 5 interview transcripts (steps 4 and 5). The final codebook (version 2.0) (Additional file 1) contained:

A) Codes to identify text fragments that provided information on the professionals’ definition of health and the health status of the residents. During the coding process the two initial codes (‘health definition’ and ‘health status’) were amended by two new, *bottom-up,* codes, ‘health behaviour ‘ and ‘action for health’, both frequently mentioned by the professionals.
B) Codes to identify text fragments that provided information on the professionals’ perceptions of neighbourhood assets (based on the Egan model [21]).
C) Codes to identify text fragments referring to individual capabilities. For these, we used Nutbeam’s model of health literacy describing the ability to find, understand, and apply knowledge that is needed to maintain one’s health [23] as this seemed a useful model to identify residents’ individual capabilities as they were described by the professionals.
D) As we found that professionals frequently referred to *unavailable* health assets we created additional *bottom-up* codes to identify neighbourhood ‘assets’ and ‘deficits’.

Two codeurs simultaneously coded all interview transcripts into Atlas.ti using the codebook and adapting it during the process as described. Differences in coding were adapted based on mutual agreement. A list of 23 unresolved items was submitted to a third researcher for final coding decisions (Fig. 1, step 2–6).

Various structured code-output reports (by code or code combinations, by code family, by respondent) as well as analytic memos drawn up during coding were used for thematic analysis (axial coding) [24] (Fig. 1, step 7–8). Finally, initial analysis results were compared to the source data, both interviews and NGT, for verification, before final analysis reports were drawn up (Fig. 1, step 9–10).

## Results

### Perceptions of health and of residents’ health status

The professionals gave several broad definitions of health. The majority of the professionals talked about health and a healthy lifestyle as a means for being able to function properly in day-to-day life and cope with problems. This was illustrated by expressions like ‘feeling good about yourself’, ‘to be able to do what you want to do’, and ‘to be happy and just have energy to get things done’. Such definitions resemble Huber’s new concept of health described before [9]; echoes of the well-known WHO definition of health as a state of physical, mental and social wellbeing were also abundant in the interviews. However, no professional explicitly named their definition as such.

In discussing health definitions more in-depth, the professionals primarily defined health as a social phenomenon. They talked frequently about people’s need to help and support others and the importance of maintaining meaningful relationships.

*"You have social bonds and you have a mutual feeling of health because, if you can do something for someone else and therefore feel valuable in society, you will also be healthier. If you can do something for someone else" (Resp 5).*

At the same time, and often even in the same sentence, they defined health as (the absence of) disorders or risk factors, as self-reported health (by residents) and as health in the sense of healthy *behaviour*: when talking about their health definition the professionals rapidly started discussing the residents’ unhealthy lifestyle.

The professionals were concerned about the residents’ health. They assessed community health as poor; referring, in particular, to obesity, loneliness and depression. They considered unhealthy lifestyles and behaviours as the main cause of this.

*“So, mental problems are abundant. And people have poor food habits; you see a lot of overweight, a lot of diabetes, a lot of high blood pressure, and many people who just have inexplicable pain. For example there is a lot of abdominal pain that cannot be explained” (Resp 8).*

The professionals did not mention any individual strengths or capacities of residents that might help them remain healthy. Instead, they focused on two individual barriers for health. First and foremost, they indicated that the residents’ unhealthy behaviour, for example with regard to nutrition and physical exercise, was caused by poverty. Secondly, they perceived a lack of knowledge or insufficient capabilities of residents, hindering healthy life styles.

*“In the supermarket, in the afternoon, you see youngsters walk around with red bull, energy drinks and potato chips. So it (healthy behaviour, LdB) all starts at home. I think many parents try, but fail; and the question is, what helps them explain to their children that this is basically unhealthy behaviour” (Resp 2).*

A number of professionals had difficulty with the language and with certain cultural views of ethnic minorities living in the neighbourhood who perceived health ‘differently’, talked about it ‘differently’ and behaved unhealthily.

*“In some cultures, for example, being overweight is an indication of status, and that you are doing well in life. At the same time, it is really detrimental to your health. Perhaps that is why people attach less importance to healthy eating and living” (Resp 4).*

### Neighbourhood health assets

Although the interview was aimed at identifying health assets, the professionals, instead, talked much about problems and barriers for health. Various professionals believed that the neighbourhood as a whole did not support the health of residents. In general, their view on the neighbourhood was quite pessimistic. They expressed a pessimistic perspective on Slotermeer.

*“The truth is that there are all kinds of factors that influence health and, in Slotermeer, they are almost all negative” (Resp 5).*

A more detailed discussion about each different dimension of the Egan model, offered a more varied perspective on the environment. The professionals interviewed identified both positive and negative aspects of the neighbourhood’s physical and social environment (Table 4). Dimensions that, according to the professionals, contained predominantly positive aspects were ‘Services’, ‘Housing and the built environment’, and ‘Transport and connectivity’. The other dimensions, ‘Governance’, ‘Environmental’, ‘Social and cultural’ and ‘Economy’ were considered to contain mostly negative aspects (or absent health assets).

**Table 4:** Identified opportunities and problems in the living environment

Neighbourhood dimensions (Egan 2004)	Asset	# Mentioned*	Deficit	# Mentioned*
*(+) Services*	Many services available	32 (13 resp)	Budget cuts	10 (7 resp)
	Accessible/adapted to community needs	21 (10 resp)	Poor link to community	7 (6 resp)
	Cooperation	7 (6 resp)		
*(+) Housing and the built environment*	Green space / Sloterplas lake	29 (15 resp)	Poor housing	18 (15 resp)
	Renovated dwellings	5 (5 resp)	Small dwellings for large families	5 (5 resp)
			Unsafe	5 (4 resp)
			insufficient green space/ clean area	4 (2 resp)
*(+) Transport and connectivity*	Public transport and connectivity	13 (13 resp)		
*(−) Governance*			No insight in public administration	7 (7 resp)
*(−) Environmental*			Rubbish in the streets	10 (10 resp)
*(−) Social and cultural*	Many activities	12 (8 resp)	Poor social cohesion	19 (11 resp)
	Culture mix (positive)	4 (4 resp)	Insufficient culture mix	13 (8 resp)
*(−) Economy*			Poverty	42 (17 resp)
			One-sided economy	8 (8 resp)
			Unemployment	7 (4 resp)
			Unhealthy food supply	5 (5 resp)

This concerns the number of times the issue was presented in the interviews. In brackets: number of professionals bringing the issue forward

Linkages between different neighbourhood dimensions, or assets/deficits appeared, for example litter in the streets (environment dimension) was linked to lack of social responsibility (social and cultural dimension). This was confirmed by code co-occurrence: text fragments were frequently coded for more than one dimension.

All professionals regarded the, in their eyes plentiful, *Services* dimension (healthcare, social activities and social services) as the most important contributor to health. This included the services they provided themselves. A few professionals devoted some of their free time to activities with local residents, for example by leading a walking group, with a view to activating residents. The professionals referred to the range of services on offer as cohesive, accessible and usable for residents. They were positive about how these services were coordinated and talked not only of their own work, but also about activities of the other professionals that they considered valuable.

*“So professionals in the neighbourhood collaborate in all kinds of different ways. And these initiatives are successful because we can easily contact each other” (Resp 11).*

At the same time, however, the professionals referred to three important barriers to appropriately deliver their services. Firstly, half of the professionals also believed that communication about the range of services linked up insufficiently with residents’ perceptions, and wanted to change that. Secondly, they pointed out that cutbacks have led to impoverished and fewer services. And finally, they referred to bureaucracy and complex regulations as a hindrance to the health of, and care for, residents.

*“…whenever I visit a group of care avoiders, one of the first things I do is help them with the administration. Because there is far too much of it and they don't know how to do it and then they receive a reminder (…) What is particularly distressing is that the human dimension has just gone. All the rules and regulations are not making it any easier” (Resp 9).*

The second most important health-enhancing dimension was *Housing and the built environment*. The local greenery, containing attractive parks and the Sloterplas - a recreational lake- was often mentioned and was mostly referred to as an important asset benefiting community health. The built infrastructure however was rated negatively. The professionals were of the opinion that the dwellings in the neighbourhood were of poor quality and too small for the size of the households living there. They also thought that not enough houses were available that were fit for residents to live in. A few professionals indicated that the built living environment was unsafe, due to inhabitable empty buildings.

*“There are a lot of empty buildings, people engage in fraudulent practices and there are cannabis plantations in garages and that kind of thing” (Resp 5).*

Thirdly, *Transport and connectivity* was a dimension that the professionals considered health enhancing. The professionals regarded the *traffic infrastructure* in Slotermeer as safe, and public transport as excellent, particularly for elderly people and people with disabilities.

Of the dimensions rated mainly as less beneficial to resident health, the *Governance* dimension was rated least negative: the professionals did not have much to say about this dimension. However, they implicitly criticised local government by stating that they did not really have an insight into how public administration actually contributed to the health of residents. They expressed a desire for more visibility of policy makers both for themselves, as well as for residents and other stakeholders. According to the professionals, this would enable them, residents and other stakeholders to better understand and respond to local health and other health-relevant policies.

For the E*nvironment* dimension, also rated negatively during the interviews, the only issue mentioned was rubbish in the streets. The professionals blamed that on residents who ‘did not understand that rubbish belongs in rubbish bins’.

Most professionals considered the *social infrastructure* in the neighbourhood to be insufficient despite the many social and cultural activities. The social cohesion in the neighbourhood was assessed as being low. According to the professionals, there was little contact between residents and residents did not take any responsibility for their neighbourhood. They considered improving the social infrastructure as a matter deserving urgent attention. Although a few described the ‘lively’ mix of cultures as positive, most professionals regarded the dividing lines between the various cultural groups of the neighbourhood as a major problem.

*“It is not at all harmonious. If I look at my own neighbourhood (=Slotermeer, Ldb) all the Turkish people live close together, as do the Moroccans, with the Dutch people ending up living somewhere else. I do not see any harmony” (Resp 7).*

Lastly, all the professionals referred to the *Economy* dimension in the neighbourhood exclusively in negative terms: a one-sided range of shops, far too many ‘unhealthy eateries’ and, above all, poverty. Professionals told us that residents were hampered in their health and healthy behaviour by unemployment, debts, insufficient money for food or other essentials and the excessive cost of medical care. They made it clear that they were personally affected by this.

*“Hardly anyone has a job. So it is quite a unique situation. There are a lot of people who are in debt management. I have to say that I sometimes find this very shocking. When I hear how little people have to survive on every month” (Resp 20).*

### How do residents use neighbourhood health assets?

The professionals indicated that, due to their poverty-stricken situations and lack of capabilities, residents made too little use of assets available in the neighbourhood. The reasons, poverty and lack of capabilities, are the same reasons that professionals gave for residents’ poor health state and unhealthy behaviour.

*"Then we also have to take account of the incomes of the people who live here, which are fairly low, so I don't think people are queueing up to register with the local gym. That is also why you only see Turkish and Moroccan women walking around the Sloterplas in the summer" (Resp 4).*

According to the professionals the residents’ low level of education also played a role because they had little knowledge about health and therefore did not make proper use of the care services provided, for example due to low levels of patient compliance, or because they did not know how to find and access the care and support they needed. In short, they felt that facilities were sufficiently available, but failed to get the residents inside. Many professionals interviewed believed that their efforts produced few results. The statements by some of them express a personal feeling of powerlessness or despondency.

*"To put it in very general terms, there is little knowledge. However, it is these people who actually have more than the average number of health-related problems. A huge number of residents barely attended primary school, for example (…) People have absolutely no idea how their body works" (Resp 18).*

The professionals talked about possible solutions and about the ways they tried to help residents overcome health barriers and to use available assets for health. They said they tried to offer services that linked up more effectively with residents’ needs. A very important solution mentioned by many professionals was to develop collective approaches, for example in the form of group consultations. A number of professionals applied this approach successfully. The professionals also regarded independent collective action of residents on health issues as a key opportunity and wanted to support this.

*“You expect a whole lot from people and some of them need real guidance. Having said that, you do see it happening. For example, groups of women get together in the neighbourhood and then you have all kinds of things going on at the same time. They have social contacts, they go on walks, they can discuss their problems and exchange experiences” (Resp. 13).*

### Results of Nominal group technique session

The results of the NGT session (Table 5) confirmed the results of the interviews. In the NGT session, the green infrastructure and the transport system were predominantly mentioned as health assets, while the social infrastructure of the neighbourhood ought to be reinforced. Lack of social cohesion was considered a key issue in this neighbourhood. The professionals participating provided two types of solutions for this lack of social cohesion: one was to more effectively use health assets in the physical environment, in particular to upgrade green spaces to become real meeting places for residents. The other, and maybe even more important solution according to professionals, was found in organising or stimulating collective and self-organisation approaches in the community.

**Table 5:** Results of NGT session: top 5 issues

Neighbourhood health asset	How this can be meaningful
Group activities for residents	Provides opportunities for physical exercise and sports and reduces loneliness. Group activities should be promoted and enhanced
Volunteers and volunteer groups	Self-organisation, as an effective approach to tackle health problems, should be stimulated
Community meeting places	Meeting places strengthen social cohesion and help reduce loneliness
Social support service point	This is needed, but currently unavailable. Residents fail to find their way to facilities and services due to poor literacy
Parks and playgrounds	These are available and can be used more effectively and intensively to improve community health

## Discussion

This study was performed to assess the perceptions of local health and welfare professionals in relation to the asset-based approach, which is advocated in the field of public health and represented in Dutch policy directions.

Firstly, our study shows that the professionals interpreted health broadly and that they emphasised the social aspects of both health and healthy behaviour, for example giving support to, or being supported by, others. However, the professionals considered the residents and their behaviour as unhealthy. They emphasised the role of poverty, unemployment and lack of education as barriers for healthy behaviour and provided many examples of this. In particular poverty was a topic that came up repeatedly, and the professionals seemed almost discouraged by the problems this caused to the residents’ health, health behaviour and (unmet) health needs. Nevertheless, they were deeply committed to the wellbeing of the residents; they tried whatever possible to assist them and help solve their problems.

Secondly, it transpired that the professionals regarded several aspects of the physical infrastructure, like greenery, as health assets, but frequently mentioned some other physical aspects, like poor housing and litter in the streets, as health barriers. The professionals considered the services provided in the neighbourhood, including their own services, as important health assets, although there were some doubts about the effectiveness and accessibility of the latter. However, in their opinion the social quality of the neighbourhood was insufficient and should be improved as a matter of urgency. When asked about the way in which residents used the existing health-related opportunities, the professionals indicated that, due to a lack of individual capacities (powerlessness and ignorance) and poverty, the residents were unable to make effective use of the existing health assets.

The perceptions of the professionals are partly supported by views of Slotermeer residents themselves. A separate study that we carried out with Slotermeer residents as ‘citizen scientists’ who interviewed fellow residents, focused on the health assets as perceived by them [25]. The residents interviewed rated the green environment in the neighbourhood as health-supporting. They also thought poverty and the poor quality of local housing posed barriers to health. The study further showed that residents felt unsafe and were annoyed by litter in public space. This links up with professionals’ views. An interesting difference comes up regarding residents’ need for information and education on health issues observed by the citizen scientists. On one hand, this matches the lack of health knowledge the professionals perceived under the residents. On the other hand, however, the residents expressed optimism: they felt that such knowledge could and ought to be transferred. Indeed, the citizen scientists themselves felt that their interview activities strengthened their own knowledge, as well as their personal abilities to take action for health. Moreover, the citizen scientists’ reported having extended their personal network and stated that discussing health would be a good way to improve the social cohesion in Slotermeer. This study was reported on by the National Institute for Public Health and the Environment. After a second round, it was again evaluated; the results will be reported in a separate paper.

The conclusion would appear to be justified that the perceptions of the Slotermeer health and welfare professionals focus more on barriers for health (of which several, like poverty and cultural differences, lie outside the health sector), than on assets, and therefore do not yet match the current Dutch policy. This confirms Dunston’s [4] observation mentioned before: implementation of a new approach in day-to-day practice does not happen by itself. As we described, the Dutch interpretation of the asset-based approach contains a strong focus on individual capacities. The professionals seemed unable to detect the residents’ individual capacities, but saw mainly inabilities. This corresponds to the findings of a Dutch study of the use of ‘strength-based’ families and children sessions, a method whereby clients themselves have to develop proposals for resolving their issues. In practice, the care providers had difficulties to mobilise their clients’ own capabilities [26]. Jansen et al. [27] argue that such ‘misfits’ have their origins in differences in the work cycles between policy and practice (and research); while in the policy cycle much depends on political opportunity, values of political parties, and a focus on broad societal challenges, in the practice cycle the focus is on creating concrete value for those in need and practical applicability. A solution may be, as Dunston suggested, to invest in development of the professionals’ capacities [4]. In addition, Jansen's solution, exchange between policy makers and professionals, may prove valuable to create a better balance between what policy makers expect and what professionals can do. Although the professionals in Slotermeer perceived few individual capacities, they did identify opportunities for resident empowerment in promoting the *combined* power of residents living in the neighbourhood. This combined power, or ‘community capacity’ is then not merely the sum of individual residents‘ capacities but a whole that is more than the sum of the parts. Moreover, the professionals themselves, being present in the neighbourhood and highly motivated to contribute to residents’ wellbeing, can be considered part of that community capacity. Reinforcing the local social infrastructure in the neighbourhood, which the professionals stated was an urgent challenge, could be more beneficial for community health than the individual approach. Moreover, the discussions based on the Egan Wheel helped to identify the interconnectedness of the different social and physical dimensions of the neighbourhood. Indeed, the national investment program for priority districts, focusing on the community level and addressing a range of aspects in that community’s environment in an integrated way, seemed promising in terms of improving community health [28]. In other words, the practice-based solutions brought forward by the professionals seem to link up well with the theoretical concepts and approaches underpinning this program.

In the introduction, we mentioned the concept of ‘positive health’. Several authors who responded to Huber’s original paper in the British Medical Journal proposing this concept (8 out of 23 responses) observed that this concept was inadequate as it does not address important health determinants nor (socioeconomic) health inequalities (http://www.bmj.com/content/343/bmj.d4163/rapid-responses). Also a more recent operationalisation of ‘positive health’, identifying six personal health dimensions(bodily functions, mental functions & perception, spiritual/existential dimension, quality of life, social & societal participation, and daily functioning) [29], does not include the impact of factors in the living environment . Our study confirms that, also from the point of view of professionals working in local practices, ‘positive health’ as it is currently defined, may seem appealing, but provides little direction for effective health promotion for low-SES groups.

The results of our study must be interpreted with a certain degree of cautiousness. After all, the group of professionals was relatively small. Having said that, it was varied in composition meaning that a more complete picture has been obtained than if only GPs or social workers had been interviewed. An important strength of the study is that it consisted of in-depth interviews yielding an abundance of information about the perspectives of these professionals. The outcomes of the interviews, confirmed by the NGT session results, also match what is already known about Slotermeer. The available quantitative data about the neighbourhood, for example the figures on loneliness (see Table 1) match with the picture of a neighbourhood with poor social cohesion.

This study focused on the perceptions of professionals. The contribution of residents is an essential element for the realisation of this approach. Their own perceptions about the health of their community were not included in the study. Additional research with residents, like the study we carried out separately, offers a good opportunity to collect the missing information. For such studies, Participatory Action Research may be an appropriate method as it has the potential to empower communities and strengthen social networks [30]. It is precisely in priority neighbourhoods such as Slotermeer, where the social quality of the living environment is below average, that such a study approach can both collect information and improve the health of residents by implementing this information in practice.

## Conclusions

Our study shows, firstly, that professionals in the priority district Slotermeer rated the health of the residents as poor and their health behaviour as inadequate. They considered poverty and lack of education as important causes of this situation. Secondly, the professionals tended to talk about barriers in the neighbourhood rather than about neighbourhood health assets. As such, it seems challenging to implement asset-based approaches. However, the professionals, based on their own experiences, did perceive the development of collective approaches as a promising direction for future community health development.

## Additional file

Additional file 1: Code book. (DOCX 21 kb)
