# Supplementary material for: Local professionals’ perceptions of health assets in a low-SES Dutch neighbourhood: a qualitative study
Source: BMC Public Health. 2017 Jul 12;18:12. doi: 10.1186/s12889-017-4555-6 (PMC5506671; doi:10.1186/s12889-017-4555-6)
Supplement: Additional file 1: — Code book. (DOCX 21 kb) [file 12889_2017_4555_MOESM1_ESM.docx]

| **Code families and codes** | **Code description** |
| --- | --- |
|  |  |
| ***Family: Health*** | |
| Health definition | Quotes about the definition of health and of health determinants |
| Health status | Quotes about residents’ health status |
| Health behaviour | Quotes about health behaviour of residents, including care services utilisation |
| Action for health | Quotes about collective activities of residents to protect, promote or improve community health |
|  |  |
| ***Family: Egan dimensions (source: Egan, 2004)*** | |
| Environmental | Quotes about the environment:   - Efficient use of resources now and in the future in the built environment and service provision - Living in a way that minimises the negative environmental impact and enhances the positive impact - Protecting and improving natural resources and biodiversity - Having due regard for the needs of future generations in current decisions and actions |
| Economy | Quotes about the local economy:   - A wide range of jobs and training opportunities - Sufficient land and buildings to support economic prosperity and change - Dynamic job and business creation - A strong business community with links into the wider economy |
| Housing and built environment | Quotes about housing and the built environment:   - Creating a sense of place - Well-maintained, local, user-friendly public and green spaces with facilities for everyone including   children and older people   - Sufficient range, diversity and affordability of housing within a balanced housing market - A high quality, well-designed built environment of appropriate size, scale, density, design and   layout that complements the distinctive local character of the community   - High quality, mixed-use, durable, flexible and adaptable buildings |
| Social en cultural | Quotes about the social environment:   - A sense of community identity and belonging - Tolerance, respect and engagement with people from different cultures, background and beliefs - Friendly, co-operative and helpful behaviour in neighbourhoods - Opportunities for cultural, leisure, community, sport and other activities - Low levels of crime and anti-social behaviour with visible, effective and community-friendly policing - All people are socially included and have similar life opportunities |
| Governance | Quotes about local governance:   - Strategic, visionary, representative, accountable governance systems that enable inclusive, active   and effective participation by individuals and organisations   - Strong, informed and effective leadership and partnerships that lead by example - Strong, inclusive, community and voluntary sector - A sense of civic values, responsibility and pride - Continuous improvement through effective delivery, monitoring and feedback at all levels |
| Transport and connectivity | Quotes about local transport and connectivity:   - Transport facilities, including public transport, that help people travel within and between communities - Facilities to encourage safe local walking and cycling - Accessible and appropriate local parking facilities - Widely available and effective telecommunications and Internet access |
| Services | Quotes about services locally offered:   - Well-educated people from well-performing local schools, further and higher education and training for lifelong learning - High quality, local health care and social services - Provision of range of accessible, affordable public, community, voluntary and private services - Service providers who think and act long term and beyond their own immediate geographical   and interest boundaries |
|  |  |
| ***Family: Health literacy (source: Nutbeam, 2008)*** | |
| Finding knowledge | Quotes about people’s abilities to find knowledge relevant to their health |
| Understanding knowledge | Quotes about people’s abilities to understand knowledge relevant to their health |
| Applying knowledge | Quotes about people’s abilities to apply knowledge to improve or sustain their health |
|  |  |
| ***Family: Asset based approach*** |  |
| Asset | Quotes about aspects that help people to remain healthy and live healthy lives |
| Deficit | Quotes about barriers for people to remain healthy and live healthy lives |
|  |  |
| ***Family: Additional codes*** |  |
| Interesting/important quotes | Quotes that are either highly illustrative for a code or code family or that provide new insights |
